# Mind Mapping in Orthodontic Education: A Two‐Cohort Action‐Research Study Exploring the Impact of Feedback on Student Learning and Perception

**DOI:** 10.1111/eje.70075

**Published:** 2026-02-12

**Authors:** Shoroog H. Agou

**Affiliations:** ^1^ Faculty of Dentistry King Abdulaziz University Jeddah Saudi Arabia

**Keywords:** concept formation, faculty guidance, feedback, integrative learning, mind mapping, orthodontics/education

## Abstract

**Introduction:**

Mind mapping is a visual learning strategy that fosters critical thinking in health professions education, yet it remains underexplored in orthodontic curricula. With varying class sizes, increasing reliance on technology, and fragmented delivery of core orthodontic concepts, there is a need for pedagogical tools that support integrative and meaningful learning. This study evaluates how instructional support significantly impacts students' perceptions and learning gains when mind mapping is introduced in an undergraduate orthodontics course.

**Materials and Methods:**

An opportunistic comparison action‐research study was conducted with two student cohorts (*n* = 187). Cohort I received structured orientation, guided instruction, and individualised feedback on their mind maps, while Cohort II completed the activity independently due to limited faculty availability. A 14‐item perception questionnaire was used to assess students' views of the mind mapping experience. Reliability testing (Cronbach's α), t‐tests, ANOVA, and regression models examined the impact of instructional support and learning styles on student perceptions.

**Results:**

The perception scale demonstrated strong internal consistency (α = 0.81). Cohort I reported significantly higher scores in 11 out of 14 perception items. Regression analysis identified cohort membership as a significant predictor of students' ability to perceive “the big picture” and recognise inter‐topic links, suggesting a positive effect of structured feedback.

**Discussion:**

The findings align with literature on feedback‐enhanced learning and highlight the value of guided visual tools in complex clinical domains. The differences between cohorts underscore the influence of faculty scaffolding on student engagement and conceptual integration.

**Conclusion:**

When embedded within structured instructional guidance, proper scaffolding, and formative feedback, learning tools can support meaningful integration of orthodontic concepts, an increasingly important safeguard against fragmented surface learning, especially in the AI era.

## 1 Introduction

Orthodontics as a discipline necessitates the ability to conceptualise the “big picture” and link ideas together [1, 2, 3]. The development of critical thinking abilities enables students to move beyond surface learning to a deeper level of analytical reasoning that supports complex orthodontic decision‐making [2, 4]. Therefore, fostering critical thinking in orthodontic training is not merely advantageous but essential to developing practitioners capable of delivering safe, strategic, and adaptive care [5]. As orthodontic education broadly evolves, there is a pressing need to shift from “learning what to think to learning how to think.”

However, translating this pedagogical tool into practice presents substantial challenges. Many pedagogical approaches, including Problem‐based learning, Case‐based Learning, and Inquiry‐based Learning [6], were introduced to help orthodontic educators develop critical thinkers [2]. Given the variability in curriculum structure, student readiness, and institutional priorities, no single instructional strategy universally fits all contexts. However, as orthodontic educators strive to develop true critical thinkers, it is useful to “zoom in” on the building‐block skills (Table 1) that underlie sophisticated critical thinking to determine the instructional strategy that best supports the learner to develop the exact critical thinking skill needed at his/her specific developmental stage [7].

**TABLE 1:** Building‐block skills to enhance critical thinking in orthodontic education: a instructional strategies selection guide.

Building‐block skill	Definition	Suggested instructional strategy
1. Clarifying and summarising information	Distilling key ideas from lectures, texts, and cases	➤ Use *Cornell notes* or *mind maps* during seminars to practice summarization and linking orthodontic concepts
2. Identifying relationships	Recognising how concepts influence or relate to each other	➤ Assign *concept map tasks* (e.g., mapping malocclusion causes and treatments)
3. Comparing and contrasting	Examining similarities and differences across approaches or cases	➤ Use *comparison tables* (e.g., growth modification vs. camouflage vs. surgery in Class II cases)
4. Applying criteria for judgement	Using clear standards to assess or choose between options	➤ Introduce *rubric‐based decision grids* in treatment planning exercises
5. Making inferences and drawing conclusions	Connecting evidence to plausible interpretations	➤ Use *case‐based discussions* where students defend diagnoses or treatment choices with evidence
6. Questioning assumptions	Identifying unexamined beliefs or routine practices	➤ Use *Socratic questioning* during clinical seminars to challenge students' reasoning
7. Reflecting on reasoning	Evaluating one's own thought process and biases	➤ Use *reflective journals* or *peer‐feedback debriefs* post‐treatment planning sessions

For example, during the early pre‐doctoral stages, the ability to distil complex material into concise summaries and then consciously reflect on those summaries to uncover connections between topics that at first glance appear unrelated is an indispensable skill, particularly when undergraduates are first exposed to the diverse topics and dimensions of orthodontics. Several tools have been proposed in the higher educational literature to help students scaffold complex concepts (Table 2). These include mind maps [8], concept maps [9], decision trees [10], flowcharts [11], and graphic organisers such as comparative tables, matrices, or inforgraphs [12]. While mind maps favour rapid, associative summarization and are excellent for personal learning and creative thinking, concept maps impose a more rigorous structure that makes them better suited to assessing and refining students' conceptual frameworks [13, 14]. Many educators use both sequentially (Table 3): start with mind mapping to generate ideas, then convert the material into a concept map to scrutinise and solidify the underlying knowledge network.

**TABLE 2:** Comparative analysis of summarization methods in education: effectiveness, acceptance, and critical thinking impact.

Method	Acceptance	Usefulness	Academic achievement impact	Critical thinking development
Mind maps	High	Excellent for visualising complex relationships and constructing the big picture	Significant	Strong
Concept maps	High	Deep understanding of complex, interconnected ideas; supports knowledge integration	Significant	Strong
Decision Trees	Moderate	Problem‐solving, procedural clarity	Moderate	Moderate
Flowcharts	High	Process mapping	Moderate	Moderate
Graphic organisers	Variable	Data comparison, structured recall	Limited	Low

**TABLE 3:** Key differences between mind maps and concept maps.

Feature	Mind map	Concept map
Origin & theoretical roots	Popularised by Tony Buzan (1970s) as a memory‐and‐creativity aid; grounded in associative thinking and dual‐coding (verbal + visual) principles.	Developed by Joseph Novak & Bob Gowin (early‐1970s) to represent Ausubel's meaningful‐learning theory; designed as a formal knowledge‐representation tool.
Overall shape	*Radial “sun‐burst”*: one central image/phrase; major branches radiate outward, then sub‐branches; looks like a tree viewed from above.	*Hierarchical network*: broad concept(s) at the top, progressively specific concepts below; cross‐links weave laterally between branches, forming a mesh.
Node content	Usually, *single key words* or very short phrases; encourages speed and divergent thinking.	Full concepts written as *propositions*; each link forms a readable statement (concept A –*linking phrase*→ concept B).
Link labels	Branches are typically un‐labelled; meaning inferred from proximity/colour/thickness.	*Linking phrases are mandatory*, e.g., “causes”, “is part of”; makes relationships explicit and testable.
Cross‐links between branches	Optional and uncommon; map remains mostly tree‐like.	Actively encouraged; quality judged partly by richness of cross‐links that reveal non‐obvious relationships.
Primary educational uses	Brainstorming, note‐taking, planning essays, summarising lectures, creativity boosting.	Diagnosing prior knowledge, assessing conceptual change, facilitating deep understanding of a concept.
Assessment potential	Scoring rubrics exist (e.g., branch count, depth), but reliability is moderate because structures are idiosyncratic.	Well‐established quantitative rubrics (total propositions, hierarchy levels, cross‐links) with higher inter‐rater reliability.
Typical software tools	iMindMap, MindMeister, XMind.	CmapTools, Lucidchart, IHMC Cmap.
Cognitive emphasis	Association, elaboration, and recall.	Organisation, differentiation, and integration of knowledge.

Several studies and meta‐analyses demonstrate that mind maps and concept maps possess a superior advantage over other summarization methods in heightening academic achievements and developing critical thinking skills [8, 11, 15, 16, 17, 18].

Mind mapping and concept mapping operationalizes sound principles of cognitive theory and neuroscience in a practical and attractive way, allowing students to visually organise and connect complex ideas [19]. It does that through its radial, non‐linear layout, which mirrors the integrative “big‐picture” thinking we want students to adopt in the context of orthodontics. This technique leverages both sides of the brain, engaging visual, logical, and creative processes, which not only boost memory retention but also provide an effective strategy to organise knowledge without compartmentalised thinking, synthesise information, and develop the higher‐order thinking skills necessary for critical analysis and problem‐solving in orthodontics.

Despite compelling evidence for the educational value of mind maps and concept maps across the health sciences, their use remains minimally investigated in dental education [2, 20, 21] and virtually unexplored in orthodontic curricula. To this date, no studies described the implementation of mind maps and concept maps in an orthodontic context. It is unclear whether students naturally develop higher order thinking skills through independent mind map construction or if structured faculty involvement, through guidance, support, and individualised feedback, is necessary to maximise learning outcomes. Without clarity on this instructional dynamic, the educational value of mind mapping risks being underutilised. This manuscript therefore seeks to bridge that gap by illustrating how these strategies were embedded in an undergraduate orthodontics course and by synthesising the insights gathered over two consecutive implementation cycles. In doing so, we show that the instrument itself is only part of the equation; equal, if not greater, importance lies in the logistics and mechanics of application. By analysing what worked, what did not, and why, we provide orthodontic educators with a practical roadmap for translating these evidence‐based tools into meaningful learning experiences for their students.

## 2 Methods

### 2.1 Study Design

This is a cross‐sectional, correlational study embedded in an undergraduate orthodontic module at the Faculty of Dentistry, King Abdulaziz University (Jeddah, Saudi Arabia). The mind mapping activity was offered to two consecutive cohorts, as an assessed learning task within the regular curriculum. This project was designed as an Action Research (AR) initiative embedded within routine educational practice, aimed at improving teaching and learning. The project was approved by the Institutional Review Board (Approval No. 88‐05‐25). All data were collected as part of routine course activities; student feedback and perception questionnaire responses were anonymized before analysis. Participation (completion of the study questionnaire) was voluntary, with implied consent provided by return of the instrument, consistent with institutional guidelines.

### 2.2 Participants

All students enrolled in the course participated by submitting their mind map assignment and by completing the evaluation surveys described below.

### 2.3 Educational Intervention

The mind mapping activity was introduced as part of a broader instructional strategy aimed at fostering critical thinking and integrative learning in undergraduate orthodontics. Students in each cohort produced a mind map on an orthodontic topic of their choice (e.g., deep bite, cross‐bite, eruption problems). Maps were evaluated using a five‐criterion rubric (Table 4). In the first year of implementation, the cohort benefited from a structured rollout of the intervention. This included an orientation session, instructional guidance, and individualised feedback provided by faculty members, which culminated in a reflective dialogue to reinforce learning. The small class size during that academic year allowed for this instructional model. In the following year, the cohort size increased substantially, and available faculty resources were limited. Consequently, while the mind mapping assignment remained part of the curriculum, it was delivered without the accompanying instructional support that had been provided previously. Students were introduced to the technique but asked to complete the activity independently, with minimal feedback. This unintentional variation in implementation across cohorts was not part of a pre‐planned comparative study design; however, it presented an organic contrast that allowed for post hoc reflection, and offering a unique opportunity to reflect on the potential impact of feedback and guided instruction on student perceptions.

**TABLE 4:** Practical mind‐map scoring rubric for an undergraduate orthodontics module.

Criterion	Score 0	Score 1	Score 2	Score 3
1. Content accuracy & coverage	Only a few orthodontic terms; major errors/omissions	Basic core terms; several inaccuracies	Mostly accurate; minor gaps	Comprehensive and error‐free coverage of all key orthodontic concepts
2. Concept links (propositions)	Nodes unconnected	Simple one‐word links connect < 25% of nodes	Clear linking phrases connect most nodes; validity generally sound	Rich, meaningful linking phrases; nearly all links are valid and explanatory
3. Hierarchical depth	Flat list; no levels	Two distinct levels	≥ Three levels that reflect logical abstraction	≥ Four well‐ordered levels showing expert‐like concept hierarchy
4. Cross‐links & integration	None	1–2 cross‐links	Several cross‐links that add explanatory value	Numerous cross‐links that reveal sophisticated integration across topic branches
5. Visual organisation & readability	Cluttered; hard to follow	Some layout issues; legible	Clear, balanced layout; colour/spacing guide the eye	Highly polished: logical flow, balanced spacing, colour‐coding and symbols that aid cognition
6. Orthodontic examples/illustrations	No clinical examples	1–2 vague or generic examples	Several specific orthodontic cases or appliance illustrations	Wide variety of precise, well‐placed clinical examples that deepen understanding

### 2.4 Instruments

Participants completed a survey that assessed their perceptions of mind mapping using a 14‐item questionnaire structured into three domains: Ease of Use (1 item), Perceived Usefulness (8 items), and Facilitating Conditions (5 items). Responses were captured on a 5‐point Likert scale ranging from 1 (strongly disagree) to 5 (strongly agree). The overall internal consistency of the instrument was strong, with a reported Cronbach's alpha of 0.81, indicating acceptable reliability. In addition, participants of one cohort also completed The Felder–Soloman Index of Learning Styles (ILS) to evaluate the effect of learning style on students' perception of the mind mapping activity. The ILS has been widely used, with reported internal consistency reliability (Cronbach's alpha) ranging from approximately 0.60 to 0.78 across these cohorts, indicating acceptable reliability for educational research and practice [22].

### 2.5 Data Collection and Handling

Surveys were administered immediately after students submitted their maps. Mean scores, standard deviations, and frequencies were computed for each perception item and domain. Independent samples *t*‐tests and ANOVA were used to compare perception scores across cohorts and gender. Pearson correlation coefficients were calculated to explore associations between perception domains and ILS. A regression model was constructed to assess the predictive value of cohort membership, gender, and learning style on perceived usefulness and perceived ability to see the “big picture” through mind mapping. All analyses were conducted using standard statistical software, with significance set at *p* < 0.05.

## 3 Results

### 3.1 Participant Profile

From a total of 190 registrants, 187 completed the perception survey in full (response rate 98.4%). The sample therefore comprised 76 students in Cohort I and 111 in Cohort II; 104 were female (55.6%) and 83 male (44.4%). Cohort I students' ILS profile distribution was: 57% visual, 55% sensing, 51% active, and 52% sequential preferences, indicating a diverse learning‐style landscape within the cohort.

Table 5 presents the descriptive and inferential statistics for the 14 perception items, the three domain scores, and the overall perception score for 187 students across two cohorts. Item means ranged from 2.75 ± 1.34 (“I prefer to do this activity on my own rather than in a group”) to 4.17 ± 0.89 (“I found mind mapping an easy way of summarizing”).

**TABLE 5:** Summary of item, domain, and overall responses to perception questions presented by cohort and gender.

Domain	Question	Cohort I mean (SD)	Cohort II mean (SD)	Female mean (SD)	Male mean (SD)	Overall mean (SD)	*p* gender	*p* cohort
		*N*	*N*	*N*	*N*	*N*		
Easy to use	I found mind mapping an easy way of summarising key concepts	4.17 (0.89) 76	3.39 (1.03) 111	3.69 (1.03) 104	3.72 (1.06) 83	3.71 (1.04) 187	0.84	< 0.01
Perceived usefulness	I found mind mapping a useful way of summarising key concepts	4.18 (0.95) 44	3.58 (1.03) 109	3.86 (1.05) 104	3.53 (1) 49	3.75 (1.04) 153	0.07	< 0.01
	Using mind mapping fits with my learning style	3.5 (1.19) 44	2.83 (1.17) 109	3.12 (1.2) 103	2.82 (1.22) 50	3.02 (1.21) 153	0.16	< 0.01
	Using mind mapping helped me see the big picture of the topic I reviewed	4.28 (0.92) 75	3.57 (1.01) 110	3.9 (1) 103	3.8 (1.08) 82	3.86 (1.03) 185	0.53	< 0.01
	using mind mapping helped me see the link between the different orthodontic topics	3.76 (1.09) 76	3.27 (1.04) 111	3.27 (1.04) 104	3.72 (1.09) 83	3.47 (1.08) 187	< 0.01	< 0.01
	I will use mind mapping to summarise other topics I'm studying	3.14 (1.15) 44	2.75 (1.07) 111	2.88 (1.08) 104	2.8 (1.17) 51	2.86 (1.11) 155	0.68	0.05
	I expect to get not less than 80% if I got examined on my topic	3.66 (0.99) 44	3.39 (1.06) 111	3.46 (1.01) 104	3.47 (1.12) 51	3.46 (1.05) 155	0.96	0.15
	I would recommend mind mapping to my colleagues	3.7 (0.91) 43	3 (1.13) 109	3.29 (1.13) 101	3.02 (1.07) 51	3.2 (1.12) 152	0.16	< 0.01
Facilitating condition	Marks were the only motive for me to do this activity	3.16 (1.1) 44	3.7 (1.04) 108	3.51 (1.08) 102	3.62 (1.1) 50	3.55 (1.08) 152	0.56	< 0.01
	I think it would have been helpful to have a lecture explaining what a mind map is	3.25 (1.22) 76	3.53 (1.1) 111	3.27 (1.17) 104	3.6 (1.13) 83	3.42 (1.16) 187	0.05	0.1
	I prefer to do this activity on my own rather than in a group	2.75 (1.34) 76	3.02 (1.15) 108	2.83 (1.14) 103	3 (1.35) 81	2.91 (1.24) 184	0.38	0.15
	Time was a major barrier in undertaking such an activity	3.77 (1.01) 73	3.58 (1.06) 111	3.55 (1.09) 103	3.78 (0.96) 81	3.65 (1.04) 184	0.14	0.22
	The feedback/tutorial were an important supplement to this homework activity	3.97 (0.88) 74	3.54 (1.06) 111	3.72 (0.94) 102	3.71 (1.1) 83	3.71 (1.01) 185	0.97	< 0.01
	In general. I found this activity useful	4.13 (0.84) 76	3.28 (1.07) 107	3.62 (1.05) 100	3.65 (1.09) 83	3.63 (1.07) 183	0.85	< 0.01
	Easy to use domain score	4.17 (0.89) 76	3.39 (1.03) 111	3.69 (1.03) 104	3.72 (1.06) 83	3.71 (1.04) 187	0.84	< 0.01
	Perceived usefulness domain score	3.92 (0.86) 76	3.2 (0.83) 111	3.39 (0.82) 104	3.61 (1.01) 83	3.49 (0.92) 187	0.12	< 0.01
	facilitating condition domain score	3.54 (0.63) 76	3.44 (0.56) 111	3.42 (0.48) 104	3.57 (0.7) 83	3.48 (0.59) 187	0.09	0.26
Overall	Overall perception	3.74 (0.64) 76	3.32 (0.63) 111	3.43 (0.57) 104	3.57 (0.77) 83	3.49 (0.67) 187	0.15	< 0.01

No significant gender difference was observed for the overall score (females = 3.43 ± 0.57, males = 3.57 ± 0.77; *p* = 0.15). Only one statistically significant gender difference emerged: males were more likely than females to agree that “mind mapping helped them see the link between different orthodontic topics” (3.72 ± 1.09 [Males] vs. 3.27 ± 1.04 [Females]; *p* < 0.01). No other items, domain scores, or the overall perception score differed by gender (*p* = 0.09–0.97). Similarly, Spearman rank correlations between each perception item/domain and ILS dimensions revealed no significant associations.

When cohorts were compared, it was observed that the first cohort reported a significantly higher overall score than the second one (3.74 ± 0.64 [Cohort I], compared to 3.32 ± 0.63 [Cohort II]; *p* < 0.01) (Table 5). In addition, students in cohort I gave consistently higher ratings than those in cohort II on 10 of the 14 items and on two of the three domain scores (all *p* < 0.01). For instance: Ease of Use averaged 4.17 ± 0.89 in Cohort I versus 3.39 ± 1.03 in Cohort II. Similarly, Perceived Usefulness averaged 3.92 ± 0.86 versus 3.20 ± 0.83.

**FIGURE 1:**
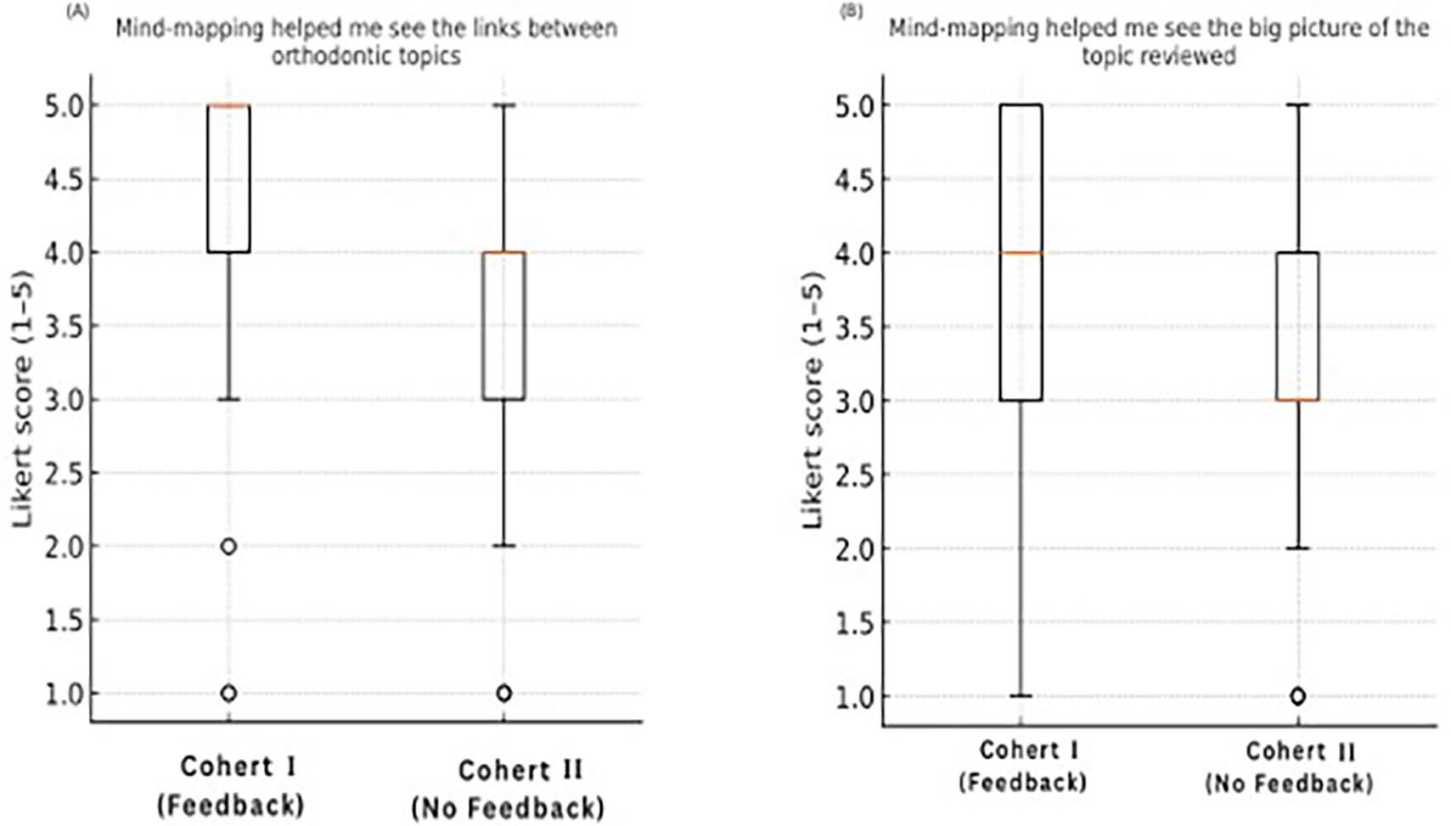
Comparison of student perceptions between Cohort I (with feedback) and Cohort II (without feedback) on two mind‐mapping outcomes: (A) “Mind‐mapping helped me see the links between orthodontic topics” and (B) “Mind‐mapping helped me see the big picture of the topic reviewed.” Boxplots with 95% confidence intervals illustrate differences in Likert‐scale responses.

One‐way ANOVA indicated pronounced cohort effects, which was confirmed by the regression model, conducted to examine the effect of cohort membership on students' perceptions of mind mapping. The outcomes: “Mind‐mapping helped me see the links between orthodontic topics,” and “Mind‐mapping helped me see the big picture of the topic reviewed,” showed a significant positive effect of belonging to the feedback cohort, accounting for approximately 11.4% and 5% of the variance, (*R* ^2^ = 0.114 and 0.050 respectively). These findings suggest that the presence of faculty guidance and feedback meaningfully enhanced students' perceived value of mind mapping as a learning tool, as shown in Figure 1.

In summary, gender and learning styles had a negligible effect on perceptions, whereas cohort membership favouring the feedback cohort was associated with consistently higher ratings of ease of use and usefulness of the mind‐mapping activity.

## 4 Discussion

This study examined the use of mind mapping as a learning tool in undergraduate orthodontic education, with a particular focus on student perceptions. While mind mapping was generally well‐received, differences between cohorts, driven by variations in implementation, shed light on the importance of faculty involvement in facilitating deep learning. The findings highlight the value of structured instructional support and feedback in maximising the educational impact of mind mapping. These results contribute to the growing body of literature on visual learning strategies in dental education and underscore the pedagogical potential of mind maps when accompanied by deliberate instructional design [20, 21].

Students' perceptions of the mind‐mapping exercise were generally favourable, reflecting the well‐documented strengths of mind‐mapping and concept‐mapping techniques in health‐professional [8, 9, 16] and dental education [23]. However, these perceptions of mind mapping appeared to be shaped more by contextual factors than by inherent traits such as learning styles or gender.

Specifically, the first cohort receiving structured orientation, guided practice, and individualised feedback, delivered using a dialogic model [24], better perceived the activity compared to the second cohort that engaged with the task independently, without real‐time support or formative input. Although this cohort effect was not part of the original experimental design, it emerged as a meaningful variable that underscores the importance of implementation. This divergence in instructional delivery appears to have had a measurable impact on students' perceptions. This finding aligns with the recent experience reported by Gil and Lee [25], who highlighted that the use of concept maps, when combined with instructor feedback, enabled students to recognise their misconceptions and enhanced their motivation to learn. Similarly, Wu and Wu indicated that faculty feedback helped students identify missed relationships, addressing the 19% gap in systematic thinking observed in self‐directed attempt [26]. These results underscore the value of guided reflection in deepening conceptual understanding, consistent with our observation that feedback played a pivotal role in shaping students' perception of mind mapping.

The difference between cohorts, while possibly attributed to other factors, is likely related to the differences in implementation between cohorts. With the sudden increase in student numbers during the second cycle, faculty were unable to replicate the support provided to cohort I, highlighting the importance of proper instructional design and the salient role of faculty support during learning activities aiming to advance thinking skills. Further, mind maps were less likely to be single‐layered, lacking hierarchical depth or cross links in the feedback cohort. An example of a student‐generated map and the rubric detailing the scoring is provided in Supporting Information, confirming that mind mapping's full educational value emerges in a feedback‐rich environment.

## 5 Lessons Learned

**FIGURE 2:**
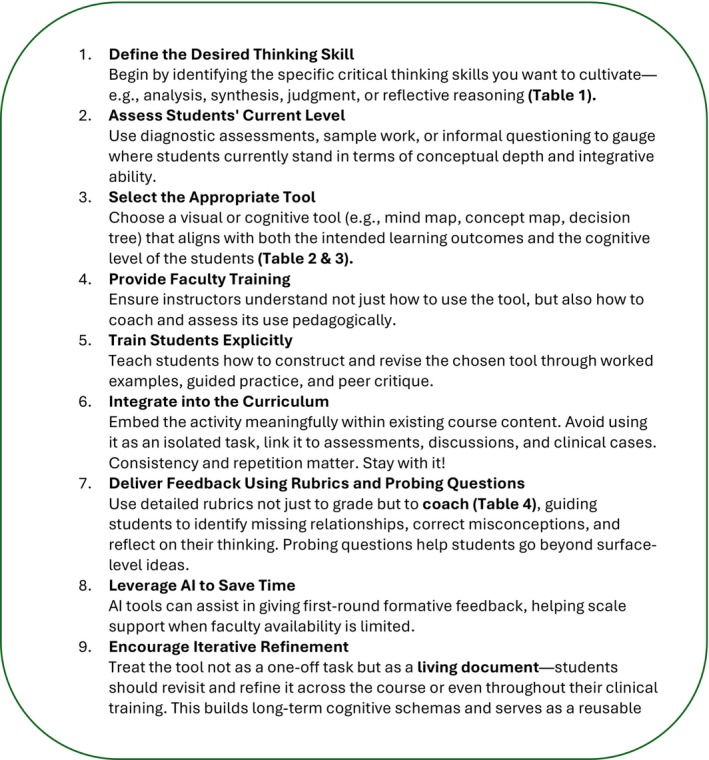
A practical roadmap for orthodontic faculty interested in fostering critical thinking skills.

This AR project yielded several core lessons for embedding mind mapping into undergraduate orthodontics: [1] mind maps deliver real pedagogical value only when nested within a robust instructional framework that supplies exemplars, probing feedback, and reflective prompts; left unguided, students revert to superficial listing [2]. Sustained faculty coaching is indispensable for pushing learners beyond surface layouts toward hierarchical, integrative representations of orthodontic knowledge [3]. Logistical realities, faculty workload, timetabling, assessment cycles need to be anticipated [4]. AI‐assisted feedback potentially offers a scalable solution, “training machines to think like faculty” and maintaining formative dialogue when human resources are stretched [5, 27]. Implementation success depends less on which tool to use and more on how it is used [6], iterative refinement is a necessity. Figure 2 distils these insights into a staged roadmap to help educators support students develop sound cognitive habits.

This study has several limitations that warrant consideration; observed differences between cohorts may have been influenced by unmeasured contextual factors such as prior academic exposure, motivation levels, or faculty–student ratios, variations in teacher facilitation and classroom dynamics. Moreover, the study relied on self‐reported perception data, which, despite showing good internal consistency, may be subject to response bias. Finally, the findings are drawn from a single institution and focused on a specific specialty within dental education, potentially limiting generalizability.

Despite these limitations, the study offers meaningful insights into how instructional design and feedback influence students' use and assessment of visual learning tools that could be generalised to other educational contexts. Future research should explore these findings using controlled, multi‐institutional designs, incorporate objective measures, and examine whether iterative use of mapping tools across courses enhances retention, critical thinking, and academic performance metrics.

## 6 Conclusion

Mind mapping proved to be an accessible, multimodal strategy for helping undergraduates deconstruct, digest, and integrate orthodontic knowledge. Yet the study also showed that without structured guidance, its impact is limited. With supportive pedagogy, including faculty‐provided feedback, mind maps have the potential to become a metacognitive lens that elevates students' cognitive structures, flags fragmented understanding and surface learning, and prompts higher‐order reasoning, which is particularly important in the AI era. Future multi‐institutional studies that triangulate perception data with objective performance and validated critical‐thinking measures will clarify the long‐term educational value of supported iterative mapping.

Even the brightest students, with the best learning tool, still need a steady hand to turn information into insight.

## Funding

The author has nothing to report.

## Ethics Statement

This study was approved by the Institutional Review Board of King Abdulaziz University (Approval No. 88‐05‐25).

## Conflicts of Interest

The author declares no conflicts of interest.

## Supporting information


**Data S1:** Supporting information.

## Figures and Tables

**FIGURE A1:**
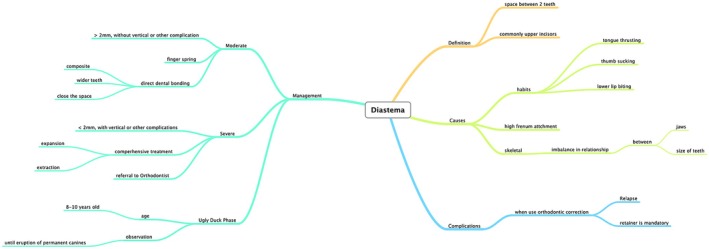
Example of a student‐generated mind map and faculty feedback (Cohort I). This figure presents an anonymized mind map submitted by a student in Cohort I, illustrating topic integration and concept hierarchy within an orthodontic theme. The map demonstrates a multilayered structure, meaningful cross‐links, and clear visual organisation, reflecting a high level of cognitive processing. Faculty feedback was provided using a structured rubric (Table A1) and targeted comments, consistent with the dialogic feedback approach implemented in that cohort.

## Data Availability

The data underlying this article will be shared on reasonable request to the corresponding author. The mind maps and analysis outputs generated during the study are available upon request for academic and non‐commercial purposes, in accordance with institutional and ethical guidelines.

## Appendix A Grading Rubric and Faculty Feedback for the Example Mind Map

**TABLE A1:** Mind‐map rubric scores and feedback for the example mind map.

Criterion	Score	Rationale & feedback
1. Content accuracy & coverage	2	The map addresses definition, aetiology, developmental stage (“ugly‐duckling” phase), management options for moderate and severe spaces, and relapse/retention. Core facts are sound, but periodontal considerations and interdisciplinary options (e.g., frenectomy and restorative closure) are omitted. Also, ugly duckling stage was mentioned under management as opposed to a possible aetiology.
2. Concept links (propositions)	1	Headings such as “Management → Moderate > 2 mm → Finger Spring” provide simple one‐word or implicit links; < 25% of nodes are connected with explanatory phrases (e.g., “because” or “leads to”).
3. Hierarchical depth	2	At least three discernible levels exist—main theme ➔ category (Causes, Management, Complications) ➔ sub‐category (habits, skeletal, bonding, extraction) ➔ list items—reflecting logical abstraction, but depth stops short of an expert four‐level structure.
4. Cross‐links & integration	0	No arrows or connective phrases bridge disparate branches (e.g., linking “high frenum” to “relapse” under complications).
5. Visual organisation & readability	1	Layout is linear with boxed headings; legible but crowded, minimal colour‐coding and no visual cues for hierarchy beyond font size.
6. Orthodontic examples/illustrations	2	Specific clinical examples such as “finger spring,” “direct composite bonding,” and “retainer is mandatory” contextualise treatment, but no diagrams or appliance sketches are included.
Feedback		“Your map demonstrates a deep understanding of the topic and good conceptual organisation. The use of cross‐links is effective, though expanding connections between aetiology and treatment planning could further enrich the integration and use of linking phrases could be improved. Excellent visual layout. Well done!”

In this study, students in Cohort I received structured, rubric‐based feedback (Table 4) to guide the development of their mind maps. An example of a student‐generated map on the topic of diastema (Figure A1) was evaluated using this rubric, with detailed scoring and commentary provided in Table A1. The initial version of the map demonstrated adequate content coverage—addressing definitions, causes, treatment strategies, and relapse considerations, but had limitations in cross‐linking and depth of integration. Notably, the “Ugly Duckling” phase was placed under management rather than considered as an etiological factor, and no visual links were drawn between high frenum and relapse. As part of the formative process, narrative faculty feedback was also given, such as: “Your map demonstrates a deep understanding of the topic and good conceptual organization. The use of cross‐links is effective, though expanding connections between etiology and treatment planning could further enrich the integration and use of linking phrases could be improved. Excellent visual layout. Well done!” Such targeted feedback, including the use of probing questions and personalised narrative comments, enabled students to iteratively revise their maps, improving visual clarity, conceptual linkage, and hierarchical depth. In several instances, this scaffolded support allowed students to transition from linear mind maps toward more structured “radial” concept maps, ultimately reinforcing deeper understanding and encouraging expert‐like integration of orthodontic knowledge. This active mentorship ensures mind maps evolve from static representations of information into living documents that reflect iterative, critical thought processes. The synergy between faculty expertise and student‐generated visual frameworks creates a scaffolded learning environment where theoretical knowledge is continuously tested, revised, and applied to clinical contexts. This interaction underscores the importance of educator engagement in mind mapping activities and lays the foundation for investigating how structured faculty support and personalised feedback directly impact the development of critical thinking competencies in orthodontic trainees.
