# Supplementary material for: Mind Mapping in Orthodontic Education: A Two‐Cohort Action‐Research Study Exploring the Impact of Feedback on Student Learning and Perception
Source: Eur J Dent Educ. 2026 Feb 12;30(3):1195–206. doi: 10.1111/eje.70075 (PMC13383303; doi:10.1111/eje.70075)
Supplement: Supplementary file 1 — Data S1: Supporting information. [file EJE-30-1195-s001.docx]

**Appendix A. *Grading Rubric and Faculty Feedback for the Example Mind Map***

**Figure: *Example of a Student-Generated Mind Map and Faculty Feedback (Cohort I)***

This figure presents an anonymized mind map submitted by a student in Cohort I, illustrating topic integration and concept hierarchy within an orthodontic theme. The map demonstrates multilayered structure, meaningful cross-links, and clear visual organization, reflecting a high level of cognitive processing. Faculty feedback was provided using a structured rubric (**Table 6**) and targeted comments, consistent with the dialogic feedback approach implemented in that cohort.

|  |
| --- |

**Table: Mind-Map Rubric Scores and feedback for the Example Mind Map**

| **Criterion** | **Score** | **Rationale & Feedback** |
| --- | --- | --- |
| **1. Content accuracy & coverage** | **2** | The map addresses definition, etiology, developmental stage (“ugly-duckling” phase), management options for moderate and severe spaces, and relapse/retention. Core facts are sound, but periodontal considerations and interdisciplinary options (e.g., frenectomy and restorative closure) are omitted. Also, ugly duckling stage was mentioned under management as opposed to a possible etiology. |
| **2. Concept links (propositions)** | **1** | Headings such as “Management → Moderate > 2 mm → Finger Spring” provide simple one-word or implicit links; < 25 % of nodes are connected with explanatory phrases (e.g. “because” or “leads to”). |
| **3. Hierarchical depth** | **2** | At least three discernible levels exist—main theme ➔ category (Causes, Management, Complications) ➔ sub-category (habits, skeletal, bonding, extraction) ➔ list items—reflecting logical abstraction, but depth stops short of an expert four-level structure. |
| **4. Cross-links & integration** | **0** | No arrows or connective phrases bridge disparate branches (e.g., linking “high frenum” to “relapse” under complications). |
| **5. Visual organization & readability** | **1** | Layout is linear with boxed headings; legible but crowded, minimal colour-coding and no visual cues for hierarchy beyond font size. |
| **6. Orthodontic examples / illustrations** | **2** | Specific clinical examples such as “finger spring,” “direct composite bonding,” and “retainer is mandatory” contextualise treatment, but no diagrams or appliance sketches are included. |
| **Feedback** |  | “Your map demonstrates a deep understanding of the topic and good conceptual organization. The use of cross-links is effective, though expanding connections between etiology and treatment planning could further enrich the integration and use of linking phrases could be improved. Excellent visual layout. Well done!” |

**Author’s Comments:**

In this study, students in Cohort I received structured, rubric-based feedback (**Table 4**) to guide the development of their mind maps. An example of a student-generated map on the topic of diastema (**Appendix A: Figure 2**) was evaluated using this rubric, with detailed scoring and commentary provided in **Appendix A: Table 6.** The initial version of the map demonstrated adequate content coverage—addressing definitions, causes, treatment strategies, and relapse considerations, but had limitations in cross-linking and depth of integration. Notably, the "Ugly Duckling" phase was placed under management rather than considered as an etiological factor, and no visual links were drawn between high frenum and relapse. As part of the formative process, narrative faculty feedback was also given, such as: “Your map demonstrates a deep understanding of the topic and good conceptual organization. The use of cross-links is effective, though expanding connections between etiology and treatment planning could further enrich the integration and use of linking phrases could be improved. Excellent visual layout. Well done!” Such targeted feedback, including the use of probing questions and personalized narrative comments, enabled students to iteratively revise their maps, improving visual clarity, conceptual linkage, and hierarchical depth. In several instances, this scaffolded support allowed students to transition from linear mind maps toward more structured “radial” concept maps, ultimately reinforcing deeper understanding and encouraging expert-like integration of orthodontic knowledge. This active mentorship ensures mind maps evolve from static representations of information into living documents that reflect iterative, critical thought processes. The synergy between faculty expertise and student-generated visual frameworks creates a scaffolded learning environment where theoretical knowledge is continuously tested, revised, and applied to clinical contexts. This interaction underscores the importance of educator engagement in mind mapping activities and lays the foundation for investigating how structured faculty support and personalized feedback directly impact the development of critical thinking competencies in orthodontic trainees.
